# Employment of 3D-Printed Bilayer Structures with Embedded Continuous Fibers for Thermal Management Applications: An Axial Cooling 4D-Printed Fan Application Case Study

**DOI:** 10.3390/polym14193952

**Published:** 2022-09-21

**Authors:** Panagiotis Zouboulis, Elias P. Koumoulos, Anna Karatza

**Affiliations:** 1 BIOG3D P.C., 1 Lavriou Ave., Technological & Cultural Park of Lavrion, 19500 Lavrion, Greece; 2 IRES—Innovation in Research & Engineering Solutions, Rue Koningin Astridlaan 59B, 1780 Wemmel, Belgium

**Keywords:** continuous fiber, 3D printing, 4D printing, shelf morphing, bilayer

## Abstract

Bi-material composite structures with continuous fibers embedded on polymer substrates exhibit self-morphing under thermal stimulus induced by the different coefficients of thermal expansion (CTE) between the two constituent materials. In this study, a series of such structures are investigated in terms of fiber patterns and materials to achieve programmable and reversible transformations that can be exploited for thermal management applications. Stemming from this investigation’s results, an axial cooling fan prototype is designed and fabricated with composite blades that passively alter their shape, specifically their curvature and twist angle, under different operating temperatures. A series of computational fluid dynamics (CFD) simulations are performed, subjecting the fan’s geometry to different flow temperatures to measure differences in airflow deriving from the induced shape transformations. Corresponding experimental trials are additionally performed, aiming to validate the simulation results. The results indicate the potential of utilizing bilayer self-morphing configurations for the fabrication of smart components for cooling purposes.

## 1. Introduction

As the complexity and density of components in modern electronic devices and assemblies increase, efficient thermal management becomes an important factor. With the trend of integration, miniaturization, and increasing power density of electronic devices, thermal management is one of the most critical challenges in current electronic packaging, which aims to dissipate the large heat flux from high-density/high-power integrated circuits and ensure high performance and long lifetime of the electronics [1]. There are several ways to manage waste heat in electronic applications, such as heat sinks or cooling fans that are focused on heat conduction to the environment [2]. Shape transformation, as a substitute for the conventional approaches based on distinct, moving surfaces, has been a subject of extensive research during the past 15 years as a promising approach to improve aerodynamic and hydrodynamic performance [3]. Initial attempts were based on deformation through the use of mechanical control structures; however, the associated weight increase from heavy and complex mechanical and hydraulic systems would frequently cancel out any efficiency advantages while also adding manufacturing complexity and reliability issues. In order to overcome these limitations, novel structural concepts and morphing solutions have been devised and analyzed in recent years, inspired by bio-mimesis or conceived by exploiting the properties of advanced materials and actuation concepts [4,5,6]. A notable example related to thermal management applications is reported, where a fan blade bonded with a layer of shape memory alloy (SMA) was modeled and tested [7]. The SMA induced deformations on the blade’s surface by activating in different temperatures of the air stream flowing around it, leading to a measurable increase in the fan’s operating efficiency. This research concluded that using bi-material configurations on cooling fan blades is indeed able to introduce a degree of passive control to the fan’s operating output. Additionally, it is reported that a mismatch in the coefficient of thermal expansion (CTE) of layers is usually used to create thermo-responsive morphing skins [8,9]. In this context, a design framework is proposed to explore the potential offered by thermally responsive materials and preprogrammed architectures with autonomous self-morphing properties and controlled bending response upon thermal stimuli (the so-called 4D printing) [10].

**Figure 1:**
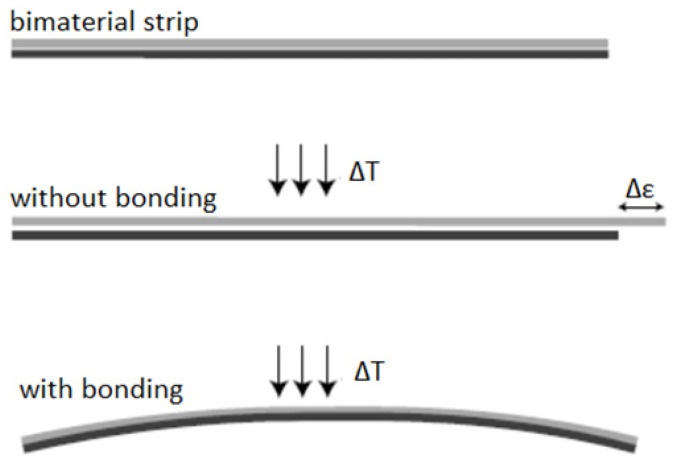
The bi-material strip concept and deformation mechanism.

Complex structure design and topology optimization offer great potential for designing 3D-printed Continuous Fiber Reinforced Polymer (CFRP) composite structures with tunable mechanical properties while maintaining lightweight potential; yet, the fiber discontinuity, length scale separation, and fiber orientation optimization of composites with complex shapes pose significant challenges along the way. Composite parts based on polymer reinforcement with continuous fibers are gaining increasing popularity in a wide range of fields due to the augmented mechanical properties of the parts combined with minimal weight values when compared to equivalent metal components. Most additive manufacturing (AM) commercial solutions that utilize continuous fiber yarns for part reinforcement are proprietary products with limited accessibility due to significantly high costs of purchase and flexibility regarding the placement of the fiber on the printed part. This deliverable’s scope focuses on the design and prototyping of a modified printhead for composite manufacturing by combining continuous fiber yarns with thermoplastic filaments in FFF processes. The advantages of the 3D-printing technology’s employment for the development of complex functional products rely on the optimal use of a wide range of customizable materials, flexible design options, and the precise production of complex, multi-material parts and components. This work is aimed toward the development of smart cooling systems that adjust their shape to optimize heat flow dynamically when subjected to different operating conditions and thus allow for the self-adjustment of convective heat transfer with the use of continuous carbon fiber printing. These approaches initially originated from the concept of the bimetallic strip, i.e., a bi-material elongated beam structure consisting of two bonded, thin metal layers (Figure 1). The strip alters its geometry under different temperature conditions due to the balance of the bending moment generated by the misfit strain against the opposing moment between its two bonded layers. The bimetallic strip can be utilized for sensing and actuating functions.

This concept is not limited to metallic materials. As long as the mechanical properties between the material couple exhibit suitable variance and appropriate inter-layer bonding, the effect can be replicated with non-metals. In this context, a dual material system was selected for the development of functioning mechanisms based on a combination of thermoplastic material bonded with continuous fiber multifilament yarns. The approach followed relies on the principle described above. Shape-Morphing Polymeric (SMPs) structures can be preprogrammed by taking full advantage of the heating process in Fused Filament Fabrication (FFF) systems since process parameters influence the phase evolution, glass transition behavior, and variation of CTE. The reinforcement of 3D-printed structures with discontinuous or continuous fibers imposes a higher driving force at the morphing behavior while at the same time increasing component mechanical properties. In both cases, preprogrammed printing paths enforce bidirectional anisotropy and unique thermal deformation in response to a thermal stimulus. The deformation of a printed structure occurs according to the deformation mechanism of the utilized material, which allows the self-deformation of dynamic objects with special functions without requiring a preprogram step. Different self-morphing mechanisms can be based on the combination of different materials with different thermal expansion coefficients and moduli of elasticity. These differences promote the shape transitions in a reversible way that, combined with design approaches, leads to controllable function.

Self-morphing properties are significantly influenced by layer thickness and layup order of the printed part. These parameters govern the meso-structure of the printed part and, therefore, the properties of the part. Among the processing parameters of additive manufacturing, printing direction is of critical importance [11]. The structure performance is also highly dependent upon the printing orientation of the part relative to the build plane, resulting in inherent anisotropy in the 3D-printed parts. In this respect, printing orientation and printing temperature were selected as constant parameters for all the 3D-printed samples in order to evaluate how deformation functionality is influenced by the design variable parameters only, since the effect of printing parameters on the pre-strain values of SMP models was investigated in many studies [11,12].

**Figure 2:**
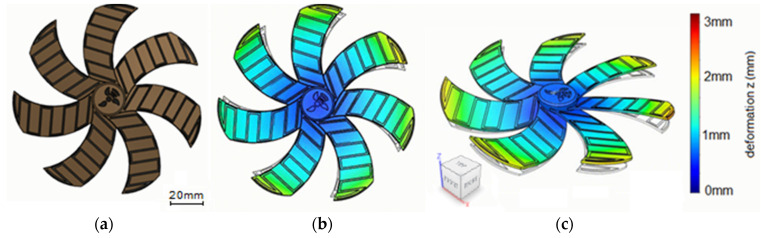
Composite fan prototype design. (**a**) Top view of the fan 3D model. (**b**) Top view of the expected deformation model. (**c**) Isometric view of the indicative expected deformation model.

**Figure 3:**
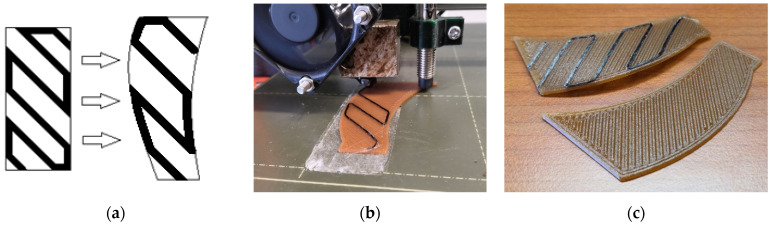
Composite blade concept. (**a**) Translation from coupon to blade geometry. (**b**) Fabrication of the composite blade. (**c**) Induced blade curvature after initial thermal treatment.

This paper examines the implementation of continuous fiber 3D-printed bilayer composites in a cooling fan prototype with composite blades of altering geometry, benefiting from their shape-morphing properties. Different fiber patterns, plastic and fiber materials, as well as blade thicknesses are investigated with the aim of selecting the optimal combination for the application. The idea behind this specific case is to develop an axial cooling fan that alters specific geometric characteristics of its blades under different temperatures, such as the pitch and curvature. This change can potentially lead to better airflow in higher temperatures without an increase in rotation speed, thus reducing the energy consumption of the cooling component. By partially or completely covering the blade’s surface using an embedded fiber pattern, a thermally induced deformation is achieved (Figure 2), which can be potentially exploited to alter the fan’s output in a temperature-dependent manner. Taking into consideration the results of the experimental deformation study, a seven-blade axial fan prototype with an external diameter of 120 mm was fabricated. Both the diameter value and the number of blades were selected to match commercially available models. The fan blades possessed embedded hatch patterns deposited diagonally with reference to the fan’s radial direction. The pattern’s orientation was chosen to induce curvature on the blades at lower temperatures, reduced gradually in higher temperatures back to a planar configuration. This initial curvature was achieved by subjecting the composite blade to a thermal treatment over the Tg of the polymer substrate and allowing it to cool down (Figure 3c). The fan prototype was fabricated using FFF 3D printing by separating the hub and blades into distinct models and subsequently assembling them using polymer adhesives. This approach facilitated the embedding of the fibers on the planar blade geometries.

## 2. Materials and Methods

### 2.1. Selected Materials

With regard to employed materials, commercial polylactic acid (PLA) filament was selected, as well as composite PLA with magnetic nanoparticle (MNP) additives. Continuous multifilament fibers were utilized for embedding in order to induce the shape-morphing effect. In the framework of this current study, both carbon and glass fibers were tested in order to determine the most appropriate candidate material for the application at hand.

Continuous multifilament fibers were utilized for embedding in order to induce the shape-morphing effect. In the framework of this current study, both carbon and glass fibers were tested in order to determine the most appropriate candidate material for the application at hand.

### 2.2. Printability Evaluation of Thermoplastic Composite Filament and Continuous Fiber Printing Assessment

**Figure 4:**
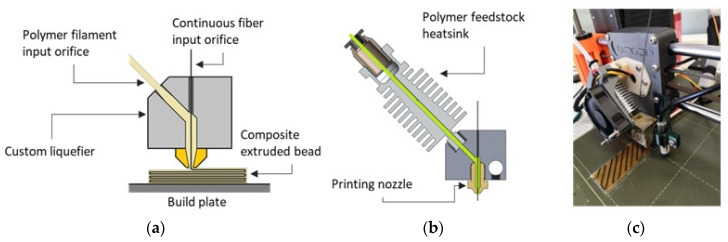
(**a**) Continuous fiber coextrusion concept. (**b**) Custom heatblock schematic. (**c**) Custom toolhead prototype continuous fiber 3D printing.

The two types of materials were combined by means of a modified fused filament fabrication (FFF) 3D-printing process based on material coextrusion through a single heated nozzle configuration (Figure 4). The fiber entered the printer’s liquefier from an upright position and was mixed in situ with the polymer filament. The plastic feedstock component was inserted via an inclined aperture. The two materials were simultaneously deposited on the build plate using customized trajectories. By removing the fiber feedstock and sealing the entry point, the printhead can be perceived as traditional FFF tooling for the creation of polymer substrates for fiber embedding.

With reference to process parameters, a printing temperature of 200 °C and printing speeds up to 30 mm/min were selected, providing optimal printing conditions for the PLA filament. Ambient humidity was kept under 30% at all times using a humidifier in order to avoid effects on the quality of the prints. With regard to the composite (fiber + polymer) feedstock, significantly lower printing speeds of up to 2 mm/s ensured proper feedstock deposition to the build plate, which was in turn heated up to 50 °C. A 0.8 mm nozzle with a corresponding 0.8 mm polymer bead and a layer height of 0.5 mm was further utilized in order to provide a suitable cross-section to facilitate the composite’s coextrusion and avoid clogging due to fiber untwisting and expanding. With regard to the extrusion of the composite material, a layer height of 0.35 mm was selected in order to ensure proper deposition and adhesion of the fiber to the plastic substrate, while in cases where fiber trajectory crossovers were expected, the layer height was marginally increased to 0.4 mm, aiming to facilitate the crossing and avoid fiber tearing that was exhibited in similar cases where smaller layer height values were applied.

### 2.3. 3D-Printed Coupons Preparation

**Figure 5:**
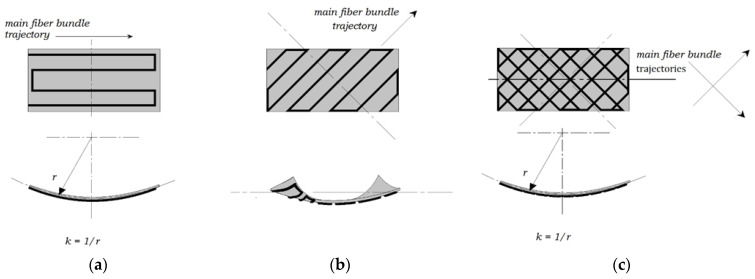
Investigated fiber patterns and expected coupon deformations. (**a**) Straight hatch along the length of the coupon. (**b**) Diagonal hatch. (**c**) Crossed hatch.

**Figure 6:**
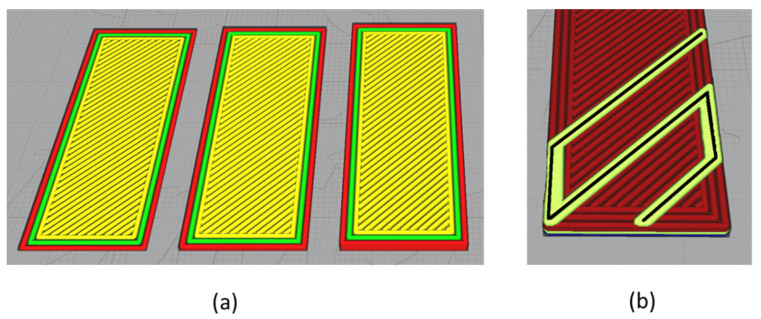
The 3D-printed substrate layout. (**a**) Number of walls and linear hatch direction. (**b**) Linear hatch and fiber direction in diagonal fiber pattern specimens.

A series of rectangular bilayer coupons were prepared for this experimental trial with a constant 50 × 25 mm size and variable thickness of 1 to 4 layers, corresponding to 0.5 to 2.0 mm. Each coupon required 8 to 15 min to fabricate, depending on the total number of substrate layers as well as the amount of embedded fiber. Three distinct embedded fiber patterns were examined, taking into consideration their expected deformations (Figure 5). In each of the three presented cases, the polymer substrate was rectangular, consisting of PLA material with different numbers of layers, two walls, and a linear hatch filling pattern placed diagonally at 45° with respect to the longitudinal direction of the coupon (Figure 6). Specifically, in the case of the diagonal fiber pattern, the linear hatch direction was selected to match the fiber trajectory in order to avoid potential deformation issues due to fiber and polymer crossover.

**Figure 7:**
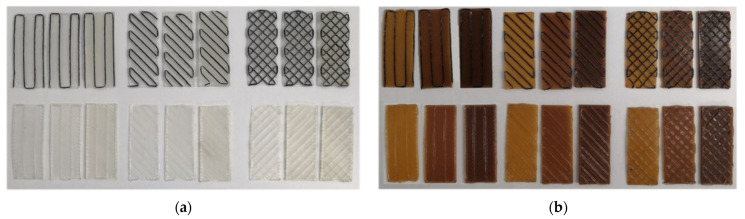
Bi-material coupons prepared for experimentation. (**a**) Commercial PLA substrate. Upper line: continuous carbon fiber; Lower line: continuous glass fiber. (**b**) Composite PLA with 1% MNP substrate. Upper line: continuous carbon fiber; Lower line: continuous glass fiber.

The parameters investigated in this stage are summarized in Table 1. Factoring the number of variable parameters, a total of 36 coupons were prepared (Figure 7).

**Figure 8:**
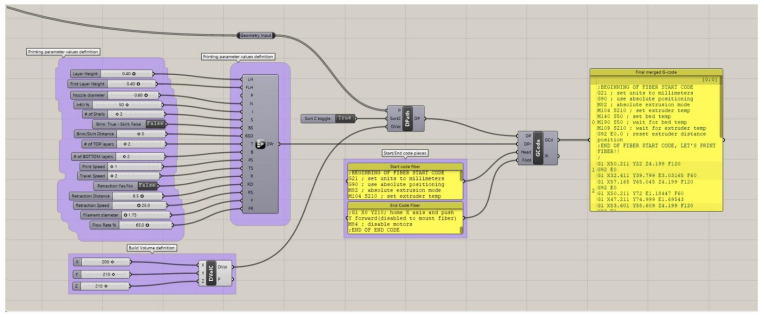
Representative parametric Grasshopper module. From left to right: printing parameter, build volume, and Start/Intermediate/End G-code pieces definition. All code pieces are joined in a single G-code file.

With regard to the design of the fiber trajectories, an alternative approach to traditional model slicing had to be adopted. The continuity of the fiber demanded single continuous toolpaths, whereas the custom patterns required tailor-made deposition trajectories. To address these requirements, a set of digital tools was assembled using the Grasshopper parametric design platform. These tools contained modules that carried out toolpath generation in both cases of polymer and composite feedstock deposition and implemented customized fiber trajectories into the printing workflow. At first, a traditional slicing module was employed for plastic feedstock extrusion without fiber in order to fabricate the coupon substrates. Following the development of the polymer constituents, an additional subcomponent was utilized to translate tailor-made continuous polyline geometries into 3D-printing G-code commands and append them to the overall G-code file. Printing parameters and starting and ending printing code pieces were defined for each module. By employing different combinations of the modules, more complex processes could be achieved by interchanging between plastic and composite extrusion multiple times. The composite toolpath generation subcomponent further supported completely 3D toolpaths, potentially allowing for fiber deposition in compliant trajectories in curved surfaces. An indicative representative depiction of a single module is depicted in Figure 8.

### 2.4. Self-Morphing Properties Assessment

**Figure 9:**
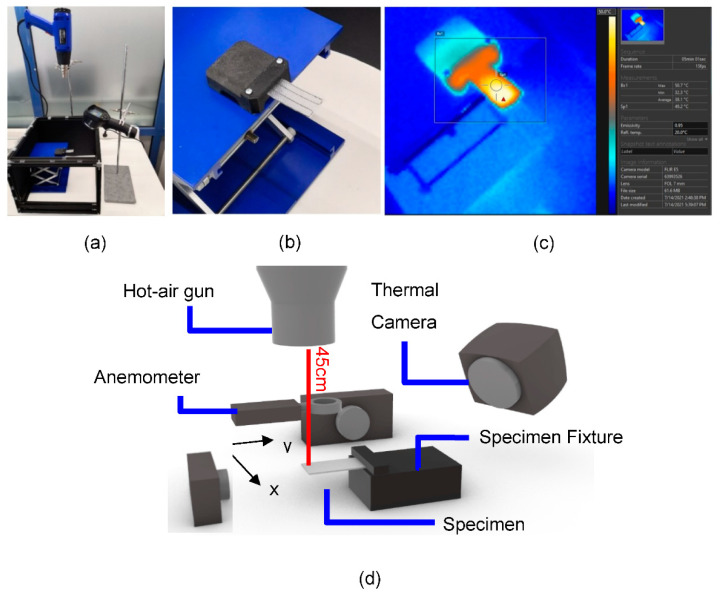
Experimental setup for shape-morphing examination. (**a**) General layout. (**b**) Coupon fixture. (**c**) Thermal camera temperature monitoring. (**d**) Experimental assembly schematic.

The setup prepared for the execution of the experimental procedure is depicted in Figure 9. The coupons were placed and fastened on a custom-made fixture, and a heat gun was mounted vertically above the specimen at a distance between 450 and 500 mm providing hot air flow for thermal treating. A thermal camera (Teledyne FLIR, Wilsonville, Portlant, USA) positioned next to the heat gun monitored the specimen’s surface temperature, indicating the start and end of every loading cycle. A set of two cameras were positioned and utilized to captivate the state and deformation of every coupon during experimentation, and an anemometer was also used to measure airflow velocity.

Each coupon underwent 3 consecutive heating and cooling cycles. Measurements were taken at distinct temperature thresholds, specifically 30 °C, 50 °C, and 80 °C. The coupons that provided the most promising deformations for the case at hand underwent an additional series of 10 consecutive cycles to further evaluate deformation repeatability. The experimental investigation described was employed to validate the design of a composite axial cooling fan prototype. The shape of the coupons was selected with the scope to be adaptable to blade geometry.

### 2.5. Fan Prototype Performance Evaluation

**Figure 10:**
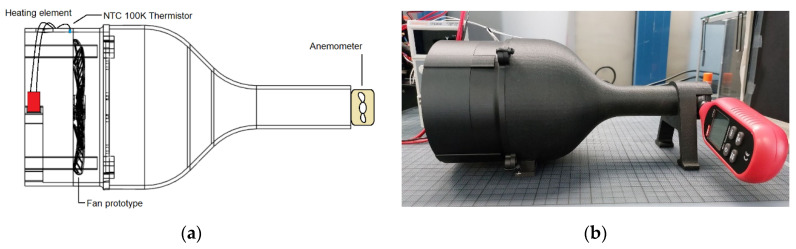
Fan experimental evaluation setup. (**a**) Schematic. (**b**) Assembled physical layout.

In order to assess the performance of the prototype comparatively to a non-composite equivalent, an additional fan was fabricated with embedding fibers on the blades omitted, resulting in a non-composite version of the same fan. An experimental layout was furthermore developed to monitor the performance of the fan under various operating conditions. This setup consisted of a fan shroud with a converging nozzle and a customized outlet housing an anemometer device (UNI-T/UT363 Anemometer, Augsburg, Germany). In the shroud’s inlet, a heating element with temperature control was positioned to provide different operating temperatures for the fan, whereas an NTC 100 K thermistor was mounted on the shroud’s wall to monitor the ambient temperature near the rotating fan. The shroud shaping took into consideration the stable transition of the flow through the fan to a laminar state (Figure 10) and the size of the anemometer measuring device. Measuring the average flow velocity through the anemometer and factoring its cross-section provides information regarding the volumetric air flow rate.

## 3. Results and Discussion

**Figure 11:**
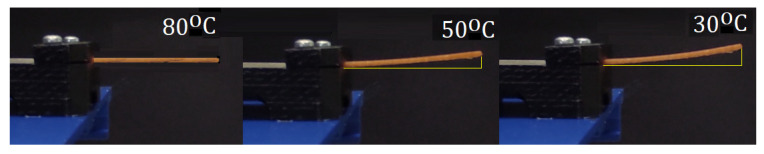
Indicative registration of coupon deflection at different temperatures.

**Figure 12:**
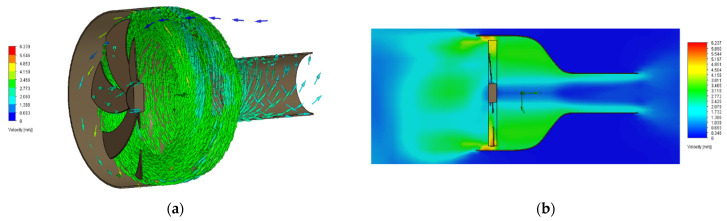
(**a**) 3D-Simulated and (**b**) cross-section of simulated airflow through the fan shroud depicting the velocity of the air moving through the shroud. Air velocity is qualitatively indicated.

**Figure 13:**
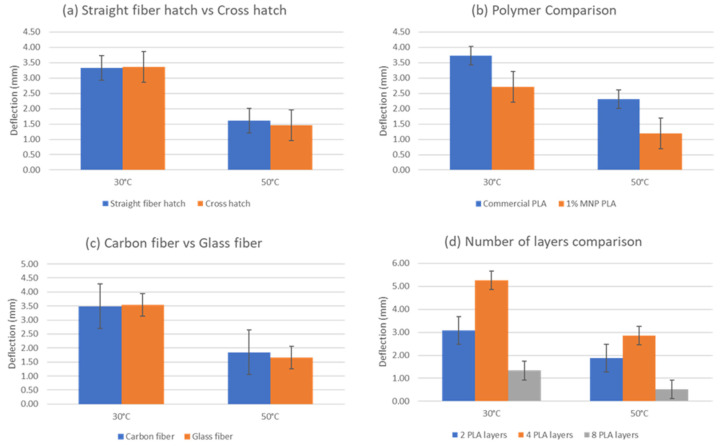
Comparative charts of deflection with regard to investigated parameters. (**a**) Straight fiber hatch pattern against crossed hatch pattern. (**b**) Behavior of the two polymer substrates, averaged for every fiber pattern. (**c**) Carbon fiber against glass fiber. (**d**) Coupon behavior with respect to number of substrate layers.

**Figure 14:**
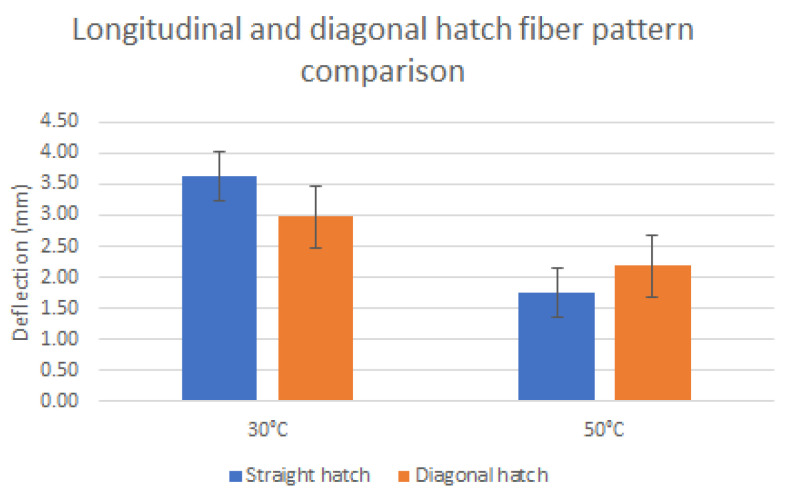
Comparative chart of deflection of diagonal vs. straight.

**Figure 15:**
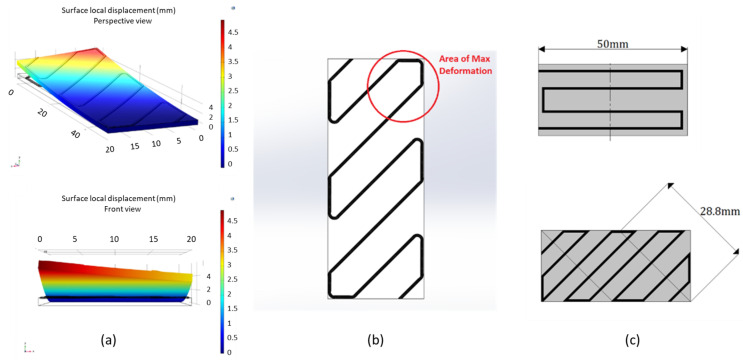
Diagonal fiber hatch coupons. (**a**) Indicative thermal stress FEA analysis deflection aiming to predict area of maximum deformation. (**b**) Area of maximum expected deformation. (**c**) Available deformation length compared to straight hatch specimens.

The experimental process to evaluate the deformation of the composite bilayer coupons directly measured their deflection distance (mm) in the designated ambient temperatures(Figure 11). Specimens composed of four polymer layers (1 mm thickness) presented, on average, the highest deflection values. Thicker specimens at 2 mm exhibited low deflection distances, especially around 50 °C, where, in some instances, the deflections were non-noticeable. Coupons at 80 °C exhibited no deflection, as is indicatively shown in Figure. This phenomenon was common in all coupons; therefore, the 80 °C category was removed from the bar charts presented in Figure 12. Each of the presented bar charts focuses on a specific feature, and deflection values occur by averaging the deformation values of every coupon that shares the feature of interest. As such, in Figure 13a, all coupons with a straight hatch are measured against coupons with a crossed hatch pattern. In Figure 13b, all specimens with a commercial polymer substrate are compared to ones with PLA with 1% MNPs. In Figure 13c, embedded carbon fiber is compared to glass fiber. In Figure 13d, all examined coupons, regardless of embedded fiber pattern or type, are grouped according to their number of substrate layers. The deflection in diagonal fiber pattern specimens is presented separately in Figure 14 in comparison to straight hatch coupons. The diagonal hatch coupons were expected to deform in a different direction from the straight and crossed hatch, following the direction of the embedded pattern. As such, a complementary FEA simulation was carried out to qualitatively identify the expected area of maximum deflection, as shown in Figure 15.

According to the analyzed measurements, straight fiber hatches presented remarkably similar results in comparison to crosshatch fiber trajectories. Although, around the 50 °C threshold, straight hatch patterns appeared to cause deformations 10% higher than their crossed hatch equivalents, the latter exceeded the former by a 1% margin at 30 °C. The commercial PLA filament exhibited steadily higher deformations than the filament with 1% MNPs, registering 48% larger average deflection distance values at 50 °C and 28% higher deformations at 30 °C. This difference potentially signifies a smaller CTE value for the composite PLA material or a higher Young’s modulus compared to its commercial counterpart. Regarding fiber bundle material, the results did not suggest a significant difference, with both fiber types exhibiting similar deformations. Nonetheless, the thickness of the polymer substrate appeared to significantly affect the deflection of the coupons, as shown in Figure 10. It seems 1 mm-thick coupons exhibit the highest deformations, whereas 2 mm coupons seem to barely deform within the specified temperature range. While achieving deformations comparable to 1 mm samples in several instances, 0.5 mm-thick test coupons did not perform as adequately overall due to several specimens failing due to overheating, therefore making the 1 mm the most appropriate thickness candidate in terms of deformation magnitude and stability.

The diagonal fiber pattern with reference to the coupon’s longitudinal direction was expected to present a comparatively lower total deflection to straight hatches due to the smaller available deformable length (Figure 15c) of the specimen in that direction [13]. As shown in the comparative chart above, although the actual difference between the two types of fiber geometries exhibited this expected behavior at 30 °C, average deformations caused by fiber bundles embedded diagonally with respect to the coupon’s length fell below straight hatch by a percentage of 22%. Nonetheless, deformations at 50 °C appeared to favor diagonal patterns presenting a higher average of 20% over straight patterns. A possible explanation regarding this phenomenon would be the direction of the 3D-printed polymer bead, as is also depicted in Figure 6. In the case of diagonal fiber bundles, the bead direction was completely parallel to the fiber pattern, conceivably facilitating geometry alteration in higher temperatures where the polymer substrate exhibits softer properties [14].

## 4. Conclusions

In this paper, a methodology for deformation programming upon thermal stimuli of 3D-printed bilayer cooling fan blades has been developed to optimize the performance of a cooling fan prototype in different temperature conditions. In this scope, thermoplastic composite filaments and embedded continuous fiber have been utilized. For blade manufacturing, a continuous fiber coextrusion process has been utilized, and the process parametrization has been conducted to ensure proper deposition and adhesion of the fiber to the plastic substrate. The 3D-printed coupon shape was selected with the scope to be adaptable to the blade geometry. A parametric Grasshopper module has been utilized for the G-code generation and fiber trajectories deposition. For the deformation evaluation, a testing setup has been prepared to captivate the state and deformation of every coupon during experimentation. The specimens were composed of different polymer layers and fiber trajectory orientations, while the deformation evaluation was based on the deflection distance of the specimens. Overall, experimental results showed that the addition of the fiber patterns on the fan blades had a temperature-related effect on the prototype’s performance. Though the average flow rate was decreased, the composite prototype exhibited more stable flow rates compared to its non-composite equivalent, with air volume passing through the composite fan decreasing at a lower rate in higher temperatures. Differences in airflow were not too high, though distinct behavior was observed. A composite fan was slightly more stable compared to a non-composite. Experimenting with different materials with varying Young’s moduli and glass transition temperatures, as well as biodegradable 3D-printable polymer compounds, could potentially lead to higher benefits and introduce a higher degree of sustainability to the self-morphing composite structures comprising the content of future work within this work’s outcome.

## Figures and Tables

**Table 1:** Experimental coupon parameters.

Polymer Material	Fiber Type	Fiber Pattern	Coupon Thickness
Commercial PLA	Carbon Fiber	Straight hatch	0.5 mm
Composite PLA with 1% MNPs	Glass Fiber	Diagonal hatch	1.0 mm
-	-	Crossed hatch	2.0 mm

## Data Availability

Not applicable.
